# A Novel Research Paradigm for Sarcopenia of Limb Muscles: Lessons From the Perpetually Working Diaphragm's Anti‐Aging Mechanisms

**DOI:** 10.1002/jcsm.13797

**Published:** 2025-04-13

**Authors:** Enhui Li, Rui Wang, Yanli Li, Xiang Zan, Shufen Wu, Yiru Yin, Xiaorong Yang, Litian Yin, Yu Zhang, Jianguo Li, Xin Zhao, Ce Zhang

**Affiliations:** ^1^ Key Laboratory of Cellular Physiology, Ministry of Education, Department of Physiology Shanxi Medical University Taiyuan Shanxi China; ^2^ Department of Neurology First Hospital of Shanxi Medical University Taiyuan Shanxi China; ^3^ The Neurosurgery Department of Shanxi Provincial People's Hospital Shanxi Medical University Taiyuan Shanxi China

**Keywords:** aging, diaphragm, limb skeletal muscles, sarcopenia, *Smox*, transcriptome

## Abstract

**Background:**

Skeletal muscle function and mass continuously decrease during aging. Most studies target limb muscles owing to their direct impact on mobility and falls risk. The diaphragm (DIA), also a type of skeletal muscle with different phenotype, has received less attention. Comparative research of the DIA and limb muscles can reveal their distinct aging characteristics. Critically, the potential endogenous anti‐aging mechanisms of DIA that may provide new insights into the mechanisms of sarcopenia in limb muscles remain scarce.

**Methods:**

Treadmill and grip tests assessed limb muscle function, while a lung function system evaluated respiratory function in both adult (6‐month‐old) and old (22‐month‐old) mice. Histological assessments evaluated muscle mass in both the DIA and tibialis anterior (TA). Transcriptome sequencing identified differentially expressed genes (DEGs) between the DIA and TA with aging. Adeno‐associated virus (AAV)‐encoding short hairpin (sh) RNA targeting gene was injected into adult mice's TA muscles to knockdown target gene level in TA, and AAV‐gene was injected into old mice's TA to overexpress target gene level.

**Results:**

Old mice displayed significantly reduced running distance (*p* = 0.0026), maximal speed (*p* = 0.0019), time to exhaustion (*p* = 0.0033) and grip strength (*p* = 0.0055) compared with adult mice, alongside TA's weight loss, decreased myofibre cross‐sectional area (CSA) and autophagy deficiency. However, lung function indicators (respiratory rate, tidal volume, minute ventilation volume, forced vital capacity and ratio of forced expiratory volume in 100 or 200 ms to forced vital capacity), as well as DIA weight and morphology remained stable in old mice. Transcriptional analysis revealed 61 DEGs, with significant upregulation or downregulation observed in TA, but without changes in DIA during aging. *Smox* (spermine oxidase) is one of the DEGs, responsible for catalysing the conversion of spermine to spermidine. It was reported that in muscle atrophy models such as limb immobilisation, fasting and denervation, *Smox'*s levels are positively correlated with muscle mass and function. Additionally, an increase in *Smox* also promotes mitochondrial biogenesis. In our study, AAV‐shSmox adult mice decreased running distance, speed and time, myofibre CSA alongside mitochondrial function, compared with controls. In contrast, old mice with *Smox* overexpression showed enhanced mitochondrial function.

**Conclusions:**

In conclusion, this study reveals aging diversities of TA and DIA, explores the sarcopenia of limb muscles based on the anti‐aging properties of DIA, which offers a novel perspective on limb sarcopenia. Our findings suggest *Smox* as a potential target for developing strategies to mitigate sarcopenia progression.

## 1 Introduction

Sarcopenia is a geriatric syndrome characterised by progressive decline of muscle functions, strength and mass, leading to serious complications with advancing age [1]. However, current studies are mostly focused on the muscles of the extremities rather than those in other regions; this is because the most prevalent and severe complications of sarcopenia, such as falls and fractures, are directly attributed to a decline of function, strength and mass in limb muscles [2]. The diaphragm (DIA), though a vital skeletal muscle responsible for respiration, has received relatively less attention in sarcopenia research. As skeletal muscles, both of limb skeletal muscles and DIA share the same origin and similar structures; however, they possess distinct functional phenotypes developed from the diverse functions. The limb skeletal muscles (motor muscles) are responsible for achieving postures and movement processes and exhibit intermittent patterns of movement and rest, namely, the intermittent working model. In contrast, the DIA operates under a perpetual‐motion model, undergoing rhythmic contraction and relaxation continuously throughout life owing to its essential role in ventilation [3].

Research on DIA has mostly concentrated on examining its structural and functional characteristics, investigating the aging processes and functional mechanisms of DIA from a perspective akin to those conducted on limb muscles studies [4, 5, 6]. There is a lack of comparative research examining the phenotypic differences between limb muscles and DIA, particularly exploring the anti‐aging properties of DIA related to its perpetual working mode, for understanding the development of sarcopenia in the limb muscles. Therefore, exploring the differential aging characteristics of DIA and tibialis anterior (TA, responsible for dorsiflexion of the foot) muscle, the phenotypic traits of DIA's perpetual contraction and potential intrinsic anti‐fatigue or anti‐aging mechanisms may provide novel theoretical bases and insights into the development of sarcopenia in limb muscles.

In the present study, we performed various experiments to validate our hypothesis: (1) compared the structural, functional and muscle mass alterations of the respiratory and limb muscles during the aging process of mice (differential aging); (2) examined the differentially expressed genes (DEGs) during the aging process of DIA and TA via transcriptome sequencing, screening out the genes that were unaltered in DIA but upregulated or downregulated in TA and verifying the gene expression levels using quantitative real‐time reverse transcriptase‐polymerase chain reaction (RT‐qPCR); (3) used transgenic technology to either overexpress spermine oxidase (*Smox*) in the TA of old mice or knock down *Smox* in the TA of young mice, to observe alterations in skeletal muscle function, muscle fibre changes via immunofluorescence and changes in related signalling pathway molecules; (4) explored the possible mechanisms of *Smox* in the aging process of skeletal muscles.

## 2 Methods

### 2.1 Animals

Six‐month‐old male C57BL/6J mice (Beijing Vital River Laboratory Animal Technology Co., Ltd.) served as adult mice models and 22‐month‐old males (Chengdu Dashuo Laboratory Animal Co., Ltd.) represented the old mice. Four mice per cage were housed (at 20°C, 40%–60% relative humidity), with constant food and water access. Both male and female mice exhibit varying degrees of decline in muscle mass, strength and function during aging. We chose male mice because, compared to females, they experience relatively stable hormone levels throughout the entire experimental period. This helps minimise the interference of hormonal variables and better control experimental conditions, as females undergo significant hormonal fluctuations during their physiological cycles [7]. We hope to focus on the basic mechanism of sarcopenia, ignore the complexity brought by gender and choose male mice as a model to simplify the experimental design, to more easily draw universally applicable conclusions.

### 2.2 Viral Inoculation of Muscle

Adeno‐associated virus 2/9 (AAV 2/9) was obtained from OBiO Technology Shanghai Corp (Shanghai, China). AAV‐encoding short hairpin (sh) RNA targeting *Smox* (shSmox) (8 × 10^10^ viral genomes) or scramble shRNA control under control of the ubiquitous cytomegalovirus (CMV) promoter was injected into adult mice's TA muscles. And AAV‐Smox or FLAG control under control of the CMV promoter was injected into old mice's TA muscles. Muscle function was evaluated 21 days post‐injection. We eliminated inter‐individual variations by performing experiments on each side of the TA muscles, with shRNA against *Smox* or AAV‐Smox in the right TA and the control virus in the contralateral TA.

### 2.3 Skeletal Muscle Function

#### 2.3.1 Grip Test

Mouse grip strength was measured using an animal grip strength metre (XR501, Xinruan, Shanghai, China) by gently pulling the mouse, and average peak force from three trials was recorded, excluding those where the grip failed [8].

#### 2.3.2 Inverted Grid Test

Strength was measured using the inverted‐hang test, where a mouse was placed in the centre of a homemade square grid (1‐cm mesh size) and slowly inverted over 2 s. The grid, held 40 cm above a cushioned box, recorded the duration that the mouse could maintain its grip until it fell [9].

#### 2.3.3 Gait Analysis

Mice's gait speed was analysed using Phenoscan suite and Runwayscan software (CleverSys, Inc.) [10]. Mice were trained to traverse a walkway from the white box to the black box without stopping, and their movements recorded for analysis while excluding pauses.

#### 2.3.4 Run‐to‐Exhaustion Test

Mice were subjected to treadmill (Panlab LE8710, USA) tests until exhaustion, with a shock grid behind. They acclimated for 5 min, running at an initial speed of 8 cm/s, which increased by 2 cm/s each minute. Time, maximum speed and distance were recorded [11].

### 2.4 Pulmonary Function Test

Respiratory muscle function was measured using a lung function detector (PFT‐MR; Shanghai Yuyan, China). Mice were anaesthetised with pentobarbital sodium (40 mg/kg), and their respiratory muscle function was monitored in tidal breathing (TB) and forced expiratory volume (FEV) mode. DIA muscles were collected for further analysis [12].

### 2.5 Lean Body Mass Composition

Animals were anaesthetised with 5% isoflurane (R510‐22; RWD, USA) and scanned using positron emission tomography‐computed tomography (PET‐CT) imaging instrument (IRIS, Inviscan SAS, Strasbourg, France) with continuous inhalation of 2% isoflurane to determine the lean mass.

### 2.6 Haematoxylin‐Eosin (H&E) Staining

Cryosections (10 μm) of frozen muscles were stained with an H&E staining kit (DH0006, Leigen, Beijing, China) and observed by microscopy.

### 2.7 Immunofluorescent Staining

Cryosections (10 μm) were incubated overnight at 4°C with primary antibodies (50 μL/cross‐section), followed by secondary antibodies. The primary antibodies used were anti‐MyhcSlow (BA‐F8; Hybridoma Bank, USA), anti‐Myhc2A (SC‐71; Hybridoma Bank, USA) and anti‐laminin (Sigma L9393). Secondary antibodies used were Alexa Fluor 488 (A21141, Life Technologies), Alexa Fluor 568 (A21124, Life Technologies) and Alexa Fluor 405 (A31556, USA). Images were captured with Tissue FAXS viewer software (TissueGnostics, Austria) and analysed using ImageJ software [13].

### 2.8 RNA‐Sequencing (RNA‐Seq) and Analysis

DIA and right TA muscles were isolated from C57BL/6 mice at ages 6 and 22 months. RNA was extracted via TriZol, then sequencing on Illumina novaSeq 6000 platform [14]. Differential gene expression analyses employed the DESeq R language package (v1.0.12), |log_2_ fold change| > 1, *p* < 0.05 and FPKM > 1 were considered as candidate genes.

### 2.9 RT‐qPCR

RT‐qPCR was performed using a LightCycler 96 system (Roche, USA). *Smox* RNA levels were analysed following the instruction of RNAiso Plus (Cat#9108, TaKaRa, Japan), PrimeScript^TM^ RT reagent Kit with gDNA Eraser (RR047A, TaKaRa, Japan) and TB Green Premix Ex TaqTM II (RR820A, TaKaRa, Japan). The primer sequences (Takara, Japan) were as follows:*Smox*‐F, 5′‐AGCAGGGCTTCACGGATGTC‐3′; *Smox*‐R, 5′‐GCCATTGGCTTCTGCTAGTTG; *Gapdh*‐F, 5′‐TGTGTCCGTCGTGGATCTGA‐3′; *Gapdh*‐R, 5′‐TTGCTGTTGAAGTCGCAGGAG‐3′. All primer followed the same cycling conditions: 30 s at 95°C, 40 cycles of 5 s at 95°C, 20 s at 60°C and 15 s at 65°C. Relative expression = 2 ^−(Ct, Old Smox − Ct, Old Gapdh) − (Ct, Adult Smox − Ct, Adult Gapdh)^.

### 2.10 Western Blotting

Muscle samples processed via sodium dodecyl sulphate–polyacrylamide gel electrophoresis, transferred to membranes, blocked and probed with antibodies:FLAG (AE005, ABclonal); β‐actin (AC006, ABclonal); phospho‐mTOR (p‐mTOR) (Ser2448) (5536, CST); mTOR (2983, CST); phospho‐4E‐BP1 (Thr37/46) (2855, CST); 4E‐BP1 (9644, CST); MuRF1(A3101, ABclonal); Atrogin1(A3699, ABclonal); P62(A19700, ABclonal); LC3(ab192890, Abcam), detected by secondary antibody (BA1054, BOSTER) and chemiluminescence [15].

### 2.11 Myoblast Isolation, Culture, Differentiation and Immunostaining

The experimental procedure involved four steps:isolation, culture, differentiation, and immunostaining [16].

Isolation: the TA and DIA tissues from 3‐month‐old C57BL/6 mice were minced and placed in phosphate‐buffered saline (PBS). After homogenisation, enzymatic digestion with Type II collagenase was performed. Subsequently, samples were filtered, centrifuged and resuspended in myoblast growth medium (MGM). Culture: cells were cultured in MGM on Matrigel‐coated dishes for 72 h. Differentiation: myoblasts were induced to differentiate in a differentiation medium upon reaching 85–95% confluency. Most cells were mature myotubes 72 h after differentiation. Immunostaining: cells were fixed, permeabilized and blocked before incubation with primary antibodies overnight. After washing, secondary antibodies and DAPI were applied and analysed by fluorescence microscopy.

### 2.12 Metabolite Extraction and Detection

This experiment was performed by the Metabo‐Profile Biotechnology Co., Ltd. (Shanghai, China). Ten‐milligram tissues were homogenised with 30 μL sterile water and 150 μL methanol. The lysates were then centrifuged at 18000 × *g* for 20 min at 4°C. Twenty‐microliter supernatant was collected for polyamine and β‐alanine analysis.

### 2.13 Electron Microscopy

TA were harvested and fixed with 2.5% glutaraldehyde at 4°C for 30 min, rinsed with PBS thrice and then fixed with 1% osmium acid for 2 h. Samples underwent dehydration in 50%, 70%, 80%, 90%, 95%, 100%, 100% alcohols and were permeated with a 1:1 acetone embedding agent for 1 h, then incubated at 60°C for 48 h. Ultrathin sections (60–80 nm) were stained with 2% uranyl acetate and 2% lead citrate.

### 2.14 Succinate Dehydrogenase (SDH)/Cyclooxygenase (COX) Histochemistry

Frozen sections were incubated in a medium containing sodium succinate, sodium azide, tetranitro blue tetrazolium and phenazine methosulfate to evaluate SDH activity in the TA muscles. The frozen sections were incubated in a medium containing cytochrome c and 3, 3'‐diaminobenzidine at 37°C for 20 min to evaluate COX activity in TA muscles [17].

### 2.15 Statistical Analyses

All data were analysed using two‐tailed Student's *t*‐test and one‐way analysis of variance tests and presented as mean ± standard error of mean (SEM).

## 3 Results

### 3.1 Effects of Aging on Limb and Respiratory Skeletal Muscle

**FIGURE 1:**
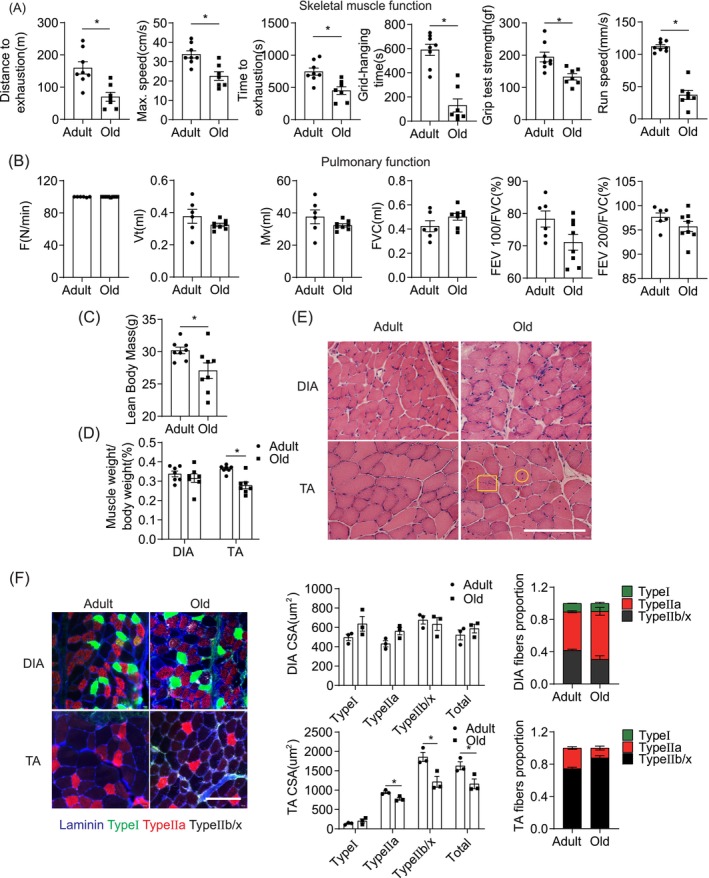
Differential aging of DIA and TA muscle. (A) The running distance to exhaustion, maximal speed, running time to exhaustion, grid‐hanging capacities, grip test strength and walking speed evaluated in 22‐month‐old mice (*n* =  7) relative to 6‐month‐old mice (*n* =  8). (B) F (respiratory rate), Vt (tidal volume), Mv (minute ventilation volume), FVC (forced vital capacity), FEV 100/FVC (ratio of forced expiratory volume in 100 ms to forced vital capacity) and FEV 200/FVC (ratio of forced expiratory volume in 200 ms to forced vital capacity) comparison of 22‐month‐old mice (*n* =  8) relative to 6‐month‐old mice (*n* =  6). (C) Lean mass of adult mice (*n* = 8) and old mice (n = 8). (D) Percentage of muscle weight normalised to body weight of old mice (*n* = 7) compared to adult mice (*n* = 7). (E) H&E staining of DIA and TA muscle CSA from adult mice (*n* = 3) and old mice (*n* = 3). Scale bars = 500 μm. (F) Representative pictures of MHC isotypes immunofluorescence of the DIA and TA muscle CSA from adult mice (*n* = 3) and old mice (*n* = 3). Triple‐labelling with α‐laminin (blue), α‐MHC‐I (green, Type I fibres) and α‐MHC‐IIa (red, Type IIa fibres) allows the estimation of the Type IIb/x fibres amount (black, nonlabeled). Scale bars = 100 μm. Quantification of CSA and number proportion of per muscle fibres in the DIA and TA muscle (*n* = 3 mice per group). Error bars represent the standard error of the mean (SEM). *p < 0.05. CSA, cross‐section area; DIA, diaphragm; H&E, haematoxylin and eosin; MHC, myosin heavy chains; TA, tibialis anterior.

Old mice exhibited reduced skeletal muscle performance compared to adults (Figure 1A), as evidenced by diminished running distance, maximum speed and endurance. Gait analysis revealed reduced gait speed, and the inverted grid test indicated significantly reduced hanging times. Additionally, grip strength was lower in old mice, highlighting impaired limb muscle function.

Adult and old mice were subjected to pulmonary function tests to assess the respiratory muscle function during aging. As shown in Figure 1B, six indicators were listed. Compared with adult mice, changes of most indicators in old mice were not significant, which suggested that the reduction in respiratory muscle function was less pronounced during aging.

### 3.2 Morphological Alterations of Mice Skeletal Muscles During Aging Are Muscle‐Specific

PET‐CT was first used to measure the lean body weight of the mice. Old mice exhibited lower lean body weight than adult mice (Figure 1C). Next, we selected TA and DIA to observe limb and respiratory muscles mass changes via weighing. While the DIA mass in old mice was unchanged compared to adults, the TA mass significantly decreased, indicating potential muscle atrophy (Figure 1D).

H&E staining (Figure 1E) and immunofluorescence staining (Figure 1F) revealed that the cross‐sectional area (CSA) of TA muscle fibres, particularly Type IIb/x, significantly decreased in old mice compared to adults. Conversely, DIA muscle cross‐sections remained stable, with old mice exhibiting a higher proportion of Type IIa and a lower proportion of Type IIb/x fibres.

### 3.3 Skeletal Muscle Transcriptome in DIA Differs From TA During Aging

**FIGURE 2:**
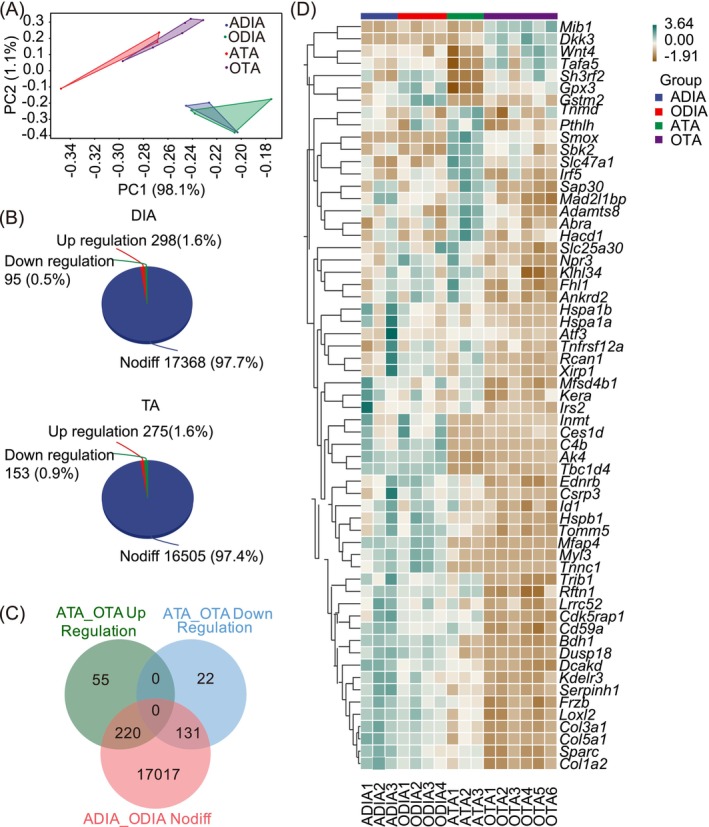
RNA‐seq of DIA and TA muscle from adult and old mice. (A) PCA plot of RNA‐seq data. (*n* = 3 in adult mice and *n* = 4–6 in old mice) (B) A pie chart indicating the genes showing expression level changes in old DIA muscle and TA muscle compared to adult muscles. (C) Comparison of DIA and TA muscle gene signatures by Venn Diagram. Pink in the Venn Diagram represents unchanged genes between adult DIA and old DIA. Green in the Venn Diagram represents upregulated DEGs in old TA muscle compared adult TA muscle. Cyan in the Venn Diagram represent downregulated DEGs in old TA muscle compared adult TA muscle. (D) Heat map of 61 key DEGs. Horizontal representation of genes: Each column represents a sample, with gene and sample name on the right and bottom sides. Green represents high expression, and yellow represents low expression. DEGs, differentially expressed genes; DIA, diaphragm; PCA, principal component analysis; RNA‐seq, RNA‐sequencing; TA, tibialis anterior.

Transcriptomic analysis of DIA and TA in aging skeletal muscles revealed significant differences. PCA distinguished adult mice TA (ATA) and old mice TA (OTA), but not between adult mice DIA (ADIA) and old mice DIA (ODIA) (Figure 2A). ODIA exhibited 17 368 unaltered genes, alongside 298 upregulated DEGs, and 95 downregulated DEGs compared to ADIA. OTA showed 16 505 unchanged genes, with 275 upregulated DEGs and 153 downregulated DEGs compared to ATA (Figure 2B). We analysed the intersection between unchanged genes in DIA and the upregulated or downregulated DEGs in TA, as shown in the Venn Diagram. Intersection analysis identified 55 genes unchanged in DIA of old mice, upregulated in TA, and 131 genes unchanged in DIA of old mice, downregulated in TA, among which 61 key genes had fragments per kilobase of transcript per million fragments mapped (FPKM) > 1 (Figure 2C,D).

### 3.4 Reduction of Smox Level Leads to Muscle Atrophy in Young Mice

**FIGURE 3:**
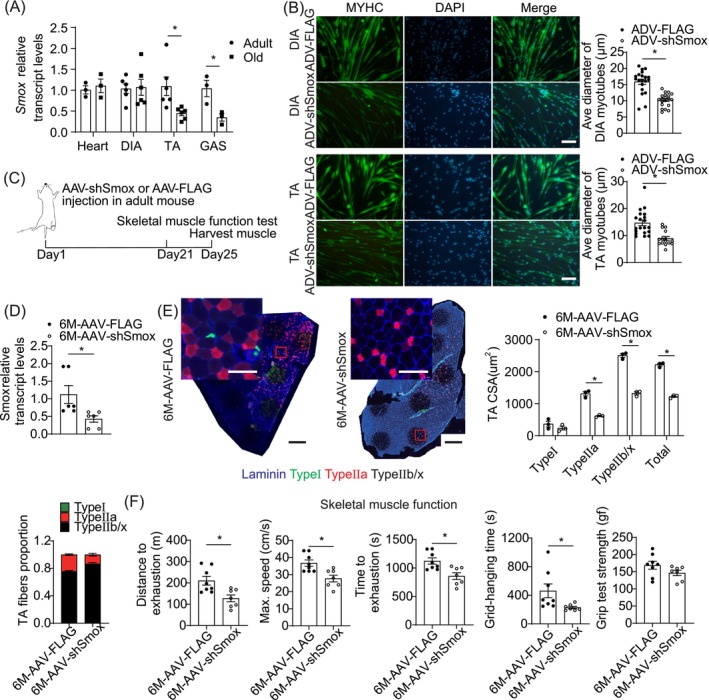
Reduction of *Smox* level‐induced muscle atrophy in young mice. (A) *Smox* expression levels in the indicated tissues of adult and old mice (*n* = 3–6 mice per group). (B) Representative images of primary myotubes treated with adenovirus‐encoding shRNA against *Smox* or control vector (*n* = 3 per group) for 48 h. Green:MYHC; blue: DAPI staining of nuclei; merge:merged images of MYHC and DAPI. Scale bars = 100 μm. (C) Protocol for TA muscles AAV2/9‐shSmox injection and associated experiments. (D) *Smox* expression levels of TA muscles injected with shRNA against *Smox* or scramble control by RT‐qPCR (*n* = 3 mice per group). (E) Immunofluorescence images of TA muscle cross‐sections in AAV2/9‐shSmox‐treated muscle and control‐treated muscle (*n* = 3 mice per group). Blue:laminin staining; green:Type I fibres; red:Type IIa fibres; black:nonlabeled. Black scale bars = 500 μm. White scale bars = 100 μm. Quantification of CSA and number proportion of per muscle fibres in TA muscles (*n* = 3 per group). (F) The running distance, maximum running speed and running time to exhaustion, hanging time and grip strength of mice TA muscles injected with shRNA against *Smox* (*n* = 7) or scramble control (*n* = 8). Error bars represent SEM. *p < 0.05. CSA, cross‐section area; DAPI, 4′,6‐diamidino‐2‐phenylindole; DIA, diaphragm; GAS, gastrocnemius muscle; MYHC, MHC, myosin heavy chains; SEM, standard error of mean; *Smox*, spermine oxidase; TA, tibialis anterior.

*Smox* (encoding SMOX) is one of these 61 key genes. Skeletal muscle atrophy correlates with a significant reduction in SMOX levels during immobilisation, fasting or denervation [18]. We investigated whether *Smox* was correlated with sarcopenia in old individuals, finding lower *Smox* expression in old TA and gastrocnemius (GAS) muscles compared to adults, whereas levels in the heart and DIA remained stable with age (Figure 3A). Myotubes differentiated from young primary DIA or TA myoblasts were infected with an adenovirus‐encoding *Smox* or a vector virus. Forty‐eight hours after infection, the myotubes were markedly thinner than those infected with the vector virus (Figure 3B). Next, the TA muscles of adult mice were locally injected with adeno‐associated virus (AAV2/9) vectors expressing shRNA against *Smox* (Figure 3C), which successfully inhibited *Smox* mRNA expression (Figure 3D). Immunofluorescence staining demonstrated that *Smox* shRNA reduced muscle fibre CSA and the percentage of Type IIa muscle fibres (Figure 3E). Mice with reduced *Smox* expression exhibited impaired motor performance, including shorter running distance, lower maximum speed, shorter running time, slower gait speed, shorter hanging time and lower grip strength (Figure 3F). These results revealed that reducing *Smox* levels impair muscle fibre size and function.

### 3.5 Overexpression of Smox Rescues Aging‐Related Muscle Atrophy

**FIGURE 4:**
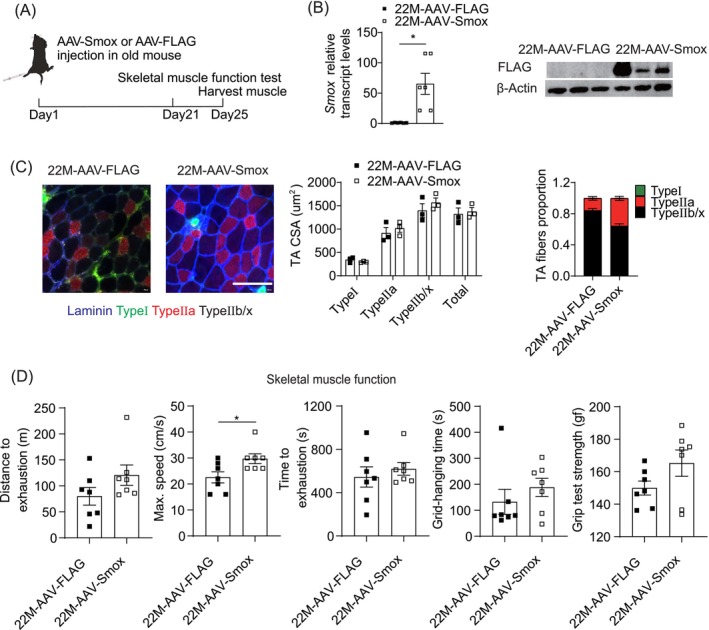
Overexpression of *Smox* rescued age‐related muscle weakness. (A) Protocol for muscle AAV2/9‐Smox injection and associated experiments. (B) Expression of *Smox* in old mice TA muscles injected with AAV2/9‐Smox or control by RT‐qPCR (*n* = 3 mice per group). Expression of FLAG in old mice TA muscles injected with AAV2/9‐Smox or scramble control by western blotting (*n* = 3 mice per group). (C) Immunofluorescence representative images of TA muscle cross sections in AAV2/9‐Smox‐treated muscle and control‐treated muscle (*n* = 3 mice per group). Blue:laminin staining; green:Type I fibres; red:Type IIa fibres; black:unlabeled. Scale bars = 100 μm. Quantification of CSA and number proportion of per muscle fibres in TA muscles (*n* = 3 per group). (D) The running distance, maximum running speed and running time to exhaustion, hanging time and grip strength of old mice TA muscles injected with AAV2/9‐Smox (*n* = 7) or control (*n* = 7). Error bars represent SEM. *p < 0.05. CSA, cross‐section area; RT‐qPCR, real‐time reverse transcriptase‐polymerase chain reaction; SEM, standard error of mean; *Smox*, spermine oxidase; TA, tibialis anterior.

Adenovirus‐encoding Flag‐tagged *Smox* and 3 × Flag were injected into the TA muscles of old mice to explore whether elevated *Smox* levels could rescue age‐related muscle atrophy (Figure 4A). AAV‐Smox increased *Smox* expression efficiently (Figure 4B), but muscle fibre size remained unchanged (Figure 4C). The percentage of Type IIa myofibres tended to be higher in TA muscles with *Smox* than those with 3 × Flag (Figure 4C). Muscle function improved with overexpression of *Smox* compared to that in muscles expressing 3 × Flag (Figure 4D). Overall, these results suggest that the overexpression of *Smox* in old muscles may rescue muscle atrophy in aging limbs.

### 3.6 Smox Improves Mitochondrial Function

*Smox* encodes SMOX, an enzyme involved in spermine catabolic process, converting spermine to spermidine and producing β‐alanine. The processes may be important mechanisms of *Smox* regulating sarcopenia. An adenovirus‐encoding shSmox was injected into one TA muscle, revealing that *Smox* knockdown significantly reduced spermidine levels compared to the contralateral muscle.

**FIGURE 5:**
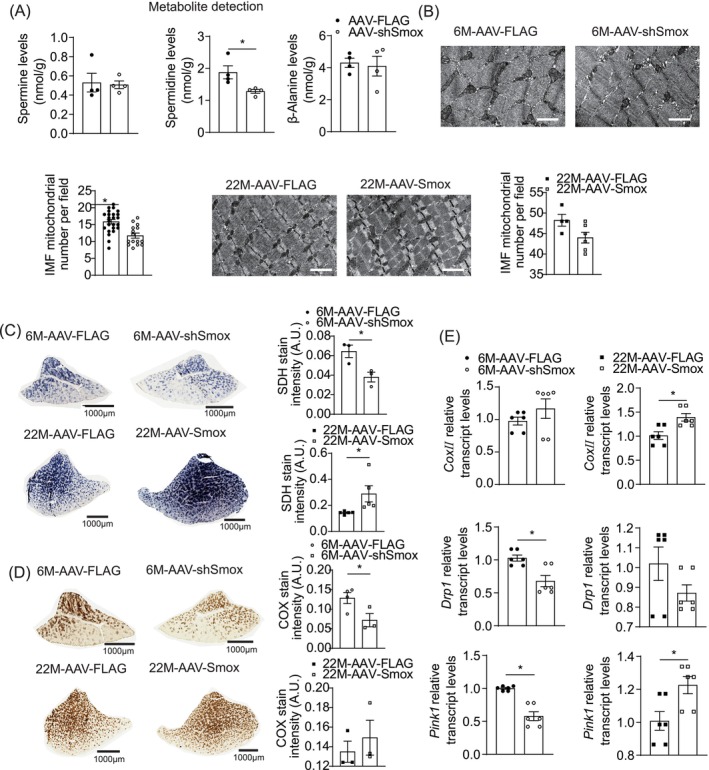
*Smox*‐mediated mitochondrial morphology and function in TA muscle. (A) Mass spectrometry‐based quantitative metabolomics analyses were performed for spermine, spermidine and β‐alanine in shSmox and scramble control TA muscles (*n* = 3 mice per group). (B–E) Representative electron micrographs, images of the COX and SDH staining of TA muscles and expression of *CoxII*, *Drp1* and *Pink1* in TA muscles by RT‐qPCR from AAV2/9/‐shSmox‐treaded muscle and control‐treated opposite side muscle of 6‐month‐old mice, AAV2/9‐Smox‐treated muscle and control‐treated opposite side muscle of 22‐month‐old mice (*n* = 3 mice per group). IMF:intramyofibrillar. White scale bars, 1 μm. Black scale bars, 1000 μm. Error bars represent SEM. *p < 0.05. COX, cyclooxygenase; *Cox II*, cytochrome c oxidase II; *Drp1*, Dynamin‐related protein 1; *Pink1*, PTEN‐induced putative kinase 1; RT‐qPCR, reverse transcriptase‐polymerase chain reaction; SDH, succinate dehydrogenase; *Smox*, spermine oxidase; TA, tibialis anterior.

Spermidine is known to enhance mitochondrial biogenesis, and dysfunctional mitochondria is a key factor in sarcopenia. Mitochondrial morphology was observed using electron microscopy. COX/SDH histochemistry was used to assess mitochondrial respiratory chain function in muscle. The number of mitochondria and the COX and SDH staining intensity severely decreased in shSmox TA muscle compared to scrambled shRNA controls. Similarly, overexpression of *Smox* led to increased mitochondrial numbers and enhanced COX/SDH staining (Figure 5B–D). These results suggested that improved mitochondrial function may be important mechanisms through which *Smox* contributes to muscle function during normal aging.

Mitochondrial quality control, including fission, fusion and mitophagy, is crucial for mitochondrial function. Dynamin‐related protein 1 (*Drp1*) regulates fission, while PTEN‐induced putative kinase 1 (*Pink1*) induces mitophagy. The shSmox TA muscle showed reduced *Drp1* and *Pink1* compared to scrambled shRNA controls in adult mice. Conversely, TA muscle overexpressing *Smox* showed increased cytochrome c oxidase II (*Cox II*) and *Pink1* (Figure 5E). These results suggest that improved mitochondrial quality control is a potential mechanism for *Smox* contributing to muscle function.

### 3.7 Smox Enhances Muscle Function by Mitigating Protein Degradation

**FIGURE 6:**
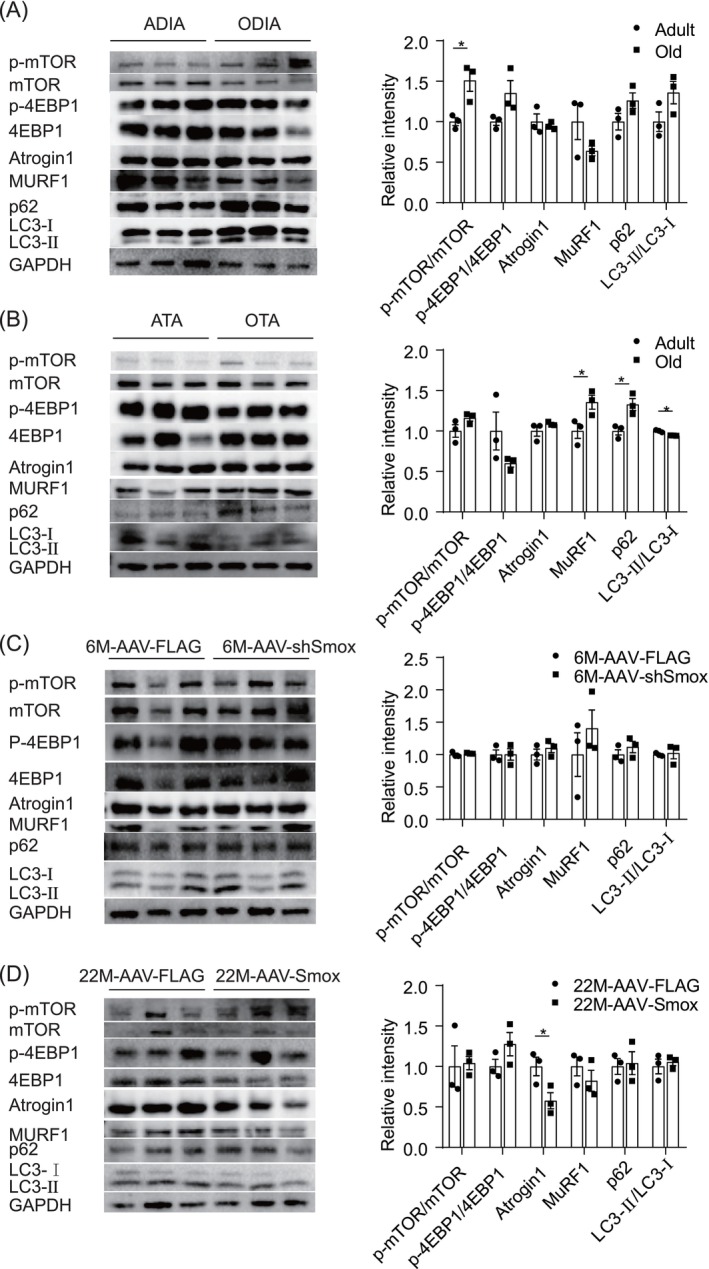
*Smox*‐mediated signalling pathway molecules involved in protein synthesis and degradation in TA muscle. Total protein extracts from DIA (A), TA (B), AAV‐shSmox (C) and AAV‐Smox (D) were immunoblotted with the indicated antibodies (*n* = 3). Quantification of Western blots by densitometric analysis on the right. Error bars represent SEM. *p < 0.05. DIA, diaphragm; *Smox*, spermine oxidase; TA, tibialis anterior.

In our investigation of signalling pathways associated with protein synthesis, we observed an increase in p‐mTOR levels in the DIA during aging, whereas no significant change was detected in the TA muscle (Figure 6A,B). Additionally, we assessed the ubiquitin‐proteasome and autophagy‐lysosome systems. The expression of the atrophy‐related ubiquitin ligase MuRF1 was elevated in OTA compared to ATA, and there was an increase in the autophagy substrate p62 and a reduction in the recruitment of LC3 on autophagosomes (LC3 lipidation, LC3II). These findings suggest that aging is associated with enhanced p‐mTOR signalling in the DIA, while in the TA, there is an increase in ubiquitin‐proteasome degradation and an impairment of autophagy, a phenomenon not observed in the aged DIA. In TA muscle with *Smox* knockdown, no significant changes were observed compared to scrambled shRNA controls in adult mice. However, TA muscle overexpressing *Smox* exhibited decreased expression of the atrophy‐related ubiquitin ligase Atrogin1 (Figure 6C,D). Collectively, these results imply that *Smox* enhances muscle function by mitigating protein degradation.

## 4 Discussion

Although both of DIA and limb muscles belong to skeletal muscles, their structural and functional characteristics exhibit a certain degree of heterogeneity. DIA performs a respiratory function, forming a permanent working mode that is never interrupted. Therefore, it is logically valid that DIA might possess endogenous antifatigue or anti‐aging mechanisms. The present study revealed the mechanisms underlying limb sarcopenia based on DIA's potential endogenous anti‐aging mechanism by comparing the differential aging of TA and DIA. This study is the first report of this creative perspective in the study of limb sarcopenia.

These results confirmed that limb muscles' structure, mass and strength decreased significantly with age through treadmill running, net hanging, gripping, gait tests, morphological detection and detection of protein molecules. However, it is doubtful so far whether DIA also undergoes the same structural and functional changes as the limb muscles during aging since the reported data on DIA are far less than those of the limb, and the existing limited studies have shown inconsistent results [13, 19, 20]. Our measurements of lung volume and ventilation in adult and old mice indicated that lung function in old mice remained stable, suggesting aging has a lower impact on DIA functions. This finding aligns with Greising et al., who noted that aging processes did not affect this ability to produce muscle force required for ventilation behaviour [21]. The decline in lean body mass in old mice, alongside unchanged wet weight in DIA, indirectly reflects the diversity of muscle mass changes. Analysis of the influence of aging on the internal structure of skeletal muscle fibres has shown that Type II fibre atrophy is an early cause of age‐related muscle atrophy [22]. Compared with adult mice, Type IIa and Type IIb/x muscle fibres CSA and the proportion of Type IIa muscle fibres showed age‐related reductions, with a corresponding decline in limb muscle function. Whereas the CSA of Type I and Type II muscle fibres controlling the ventilation behaviour increased slightly, and the proportion of Type I and Type II muscle fibres increased. Our results are consistent with previous findings that the CSA and proportion of Type I and Type II muscle fibres in mice aged 79‐week DIA were constant without obvious changes [23]. Muscle atrophy follows a reduction in cell size, primarily determined by the dynamic balance between protein synthesis and degradation. We observed an increase in the ubiquitin‐dependent atrophic protein MuRF1 in TA of aged mice, accompanied by impaired autophagy. Numerous studies have found that autophagy flux is inhibited during the aging process of limb muscles [24]. A cross‐sectional study revealed that autophagy‐related genes in the lateral femoral muscle were downregulated in elderly and frail women and positively correlated with muscle function (as assessed by the 6‐min walk test) [25]. Autophagy is a conserved catabolic process in organisms that selectively clears damaged or aging organelles and macromolecules. The long‐term presence of dysfunctional organelles in muscles may trigger catabolic pathways, ultimately leading to muscle atrophy and weakness. Proper autophagy, however, exerts a protective effect on muscles. Muscle‐specific autophagy inhibition exacerbates muscle aging phenotypes, including decreased muscle strength, mitochondrial dysfunction, oxidative stress and severe weakness. Conversely, expressing *Atg7* in skeletal muscle to activate autophagy in aged mice can enhance muscle quality [26]. However, autophagy impairment did not occur in ODIA, and the activity of the protein synthesis signalling molecule mTORC1 was increased. In line with our findings, another study compared the molecular signalling characteristics of protein synthesis and degradation in respiratory muscles (DIA and intercostal muscles) and limb muscles (gastrocnemii) of young and old rats. They discovered that autophagy deficiency was only pronounced in the gastrocnemii, not in the respiratory muscles [27]. These results suggest the presence of an endogenous anti‐aging mechanism in the DIA, potentially delaying its aging process.

Based on comparative observations of the structural and functional changes in TA and DIA during aging, transcriptome sequencing was performed to identify the genetic characteristics. Minor variations in gene expression were found between adult and old muscles, consistent with previous studies. Lee et al. reported that among 6347 genes in the gastrocnemius muscle of 5‐ and 30‐month‐old mice, only 58 (0.9%) increased and 55 (0.9%) decreased significantly with age [28]. This indicates that skeletal muscle aging is likely due to the accumulation of subtle regulatory changes rather than large‐scale gene expression alterations. We analysed aging differences specifically between DIA and TA, focusing on genes unaltered in DIA but differentially expressed in TA. A total of 428 DEGs were identified in TA, with 82% remaining stable in DIA. Analysis focused on 61 genes that were unique to TA, which is theoretically the biological basis and key factor for the differential aging of TA and DIA. In other words, understanding these transcriptional products might provide important insights into the age‐related degeneration of TA muscles. Among these 61 genes, most (48) were downregulated, indicating a global decline in activities in older people. These genes related to the extracellular matrix, such as *Sparc* and *Col1a2*, which are widely distributed throughout muscle tissue and vital for muscle growth, development, contractile force transmission, regeneration and repair [29, 30]. Additionally, key genes like *Irs2* and *Dkk3* are involved in skeletal muscle growth and atrophy [31, 32]. Understanding these transcriptional changes could shed light on age‐related degeneration in TA muscles.

An important finding of this study was that *Smox* expression was downregulated during TA aging. SMOX is a key enzyme in polyamine catabolism and converts spermine into spermidine. SMOX/*Smox* is confirmed to be associated with skeletal muscle differentiation and atrophy, inhibiting muscle atrophy by upregulating antiatrophic genes while downregulating atrophy‐related genes; knocking down *Smox* causes muscle atrophy [18, 33]. However, its role in skeletal muscle structure and function during natural aging remains unexplored. Our results indicate that *Smox* expression in the TA and GAS declines with age, whereas the DIA shows no significant changes. However, overexpression of *Smox* did not enhance TA function or quality in old mice, indicating that single‐gene regulation may not reverse compromised skeletal muscle function. In adult mice, *Smox* knockdown accelerated TA aging, which confirms this hypothesis and is consistent with previous reports. While our study elucidates the relationship between *Smox* and TA aging, we did not examine its effects on DIA due to its unique location. This research aims to identify differential genes involved in aging processes of DIA and TA, with further studies needed to explore why *Smox* levels in DIA remain unchanged during aging.

Our study identifies *Smox* as a crucial regulator of sarcopenia, highlighting its role in improving mitochondrial quality control and function to delay muscle aging. Previous research indicates that disrupting genes related to mitochondrial quality control accelerates muscle aging. Notably, mitochondrial quality control defects in the GAS of old mice are not observed in respiratory muscles, suggesting its preservation there [27]. Our results showed that mitochondrial density, COX and SDH activities and mitochondrial quality control gene expression decreased after the interference of *Smox* expression in the TA of young mice. Conversely, overexpressing *Smox* in old mice enhances the expression these genes and improves mitochondrial function. This suggests that *Smox* mitigates skeletal muscle aging through better mitochondrial quality control. Additionally, studies reveal that spermidine can prolong life and delay tissue aging [34]. In skeletal muscle, spermidine paired with exercise counteracts β‐galactosidase‐induced atrophy, improve mitochondrial quality and reduce mitochondrial swelling [35]. Old mice treated with spermidine exhibit increased mitochondrial numbers, improved arrangements and greater shape diversity. Spermidine stimulates mitochondrial autophagy and biogenesis, correlating with improved cardiac function and longevity [36]. Therefore, *Smox* may improve mitochondrial quality control by increasing spermidine levels. In summary, by comparing differential aging in TA and DIA, we explored the anti‐aging mechanism of DIA, which may provide a new theoretical basis for understanding limb sarcopenia. In other words, we investigated sarcopenia of limb muscles based on the anti‐aging properties of DIA. The current study presents a novel paradigm in this field and sheds light on the mechanisms underlying sarcopenia. The conclusion that *Smox* plays an important role in the development of sarcopenia provides novel clues and pathways for formulating strategies to delay sarcopenia.

### 4.1 Limitations

Regrettably, we did not employ a specific promoter to conduct muscle cell‐specific expression experiments. This may limit the in‐depth exploration and the derivation of more definitive conclusions. However, as we know that the TA muscle is primarily composed of muscle cells (fast‐twitch and slow‐twitch fibres), as well as fibro‐adipogenic progenitor cells, endothelial cells, immune cells, smooth muscle cells and satellite cells. These cells collectively maintain muscle physiological functions during the aging process [37, 38, 39]. Our experiments have shown that the expression of *Smox* in the TA muscle decreased with aging and was associated with functional decline. In our intervention experiments, we used the CMV promoter instead of a muscle fibre‐specific promoter to target the overall TA muscle tissue and regulated *Smox* expression. The results demonstrated that AAV‐shSmox or AAV‐Smox driven by the CMV promoter significantly affected *Smox* expression and muscle functions. The findings that artificially manipulating *Smox* expression affected muscle function are consistent with our initial hypotheses and can be logically explained. Moreover, this process more closely aligns with the functional regulation mode of the overall muscle tissue under physiological conditions.

## Ethics Statement

All the animal studies in this article were approved by the Shanxi Medical University (Ethics Approval Number: SYDL2024011).

## Conflicts of Interest

The authors declare no conflicts of interest.

## Supporting information


**Data S1** Supporting information.

**Data S2** Supporting information.

## Data Availability

RNA‐seq data have been uploaded to the National Genomics Data Center with accession number PRJCA036114.
